# Longitudinal Relationship between the Percentage of Energy Intake from Macronutrients and Overweight/Obesity among Chinese Adults from 1991 to 2018

**DOI:** 10.3390/nu16050666

**Published:** 2024-02-27

**Authors:** Xiaorong Yuan, Yanli Wei, Hongru Jiang, Huijun Wang, Zhihong Wang, Mengru Dong, Xiaohui Dong, Jiguo Zhang

**Affiliations:** 1 National Institute for Nutrition and Health, Chinese Center for Disease Control and Prevention, Beijing 100050, China; 2 Key Laboratory of Trace Element Nutrition of Health Commission of China, Beijing 100050, China

**Keywords:** macronutrients, obesity, protein, fat, carbohydrate

## Abstract

To investigate the prospective relationship between macronutrient intake and overweight/obesity, data were collected in the China Health and Nutrition Survey (CHNS) from 1991 to 2018. Adults who participated in at least two waves of the survey and were not obese at baseline were selected as the study subjects. A total of 14,531 subjects were finally included with complete data. Overweight/obesity was defined as a body mass index (BMI) ≥ 24.0 kg/m^2^. The generalized estimating equation (GEE) was used to analyze the relationship between the percentage of energy intake from macronutrients and BMI and overweight/obesity. The percentages of energy intake from protein and fat showed an increasing trend (*p* < 0.01), and the percentage of energy intake from carbohydrate showed a decreasing trend (*p* < 0.01) among Chinese adults between 1991 and 2018. Adjusting for covariates, the energy intake from fat was positively correlated with BMI, while the energy intake from carbohydrates was negatively correlated with BMI. The percentage of energy intake from non-high-quality protein and polyunsaturated fatty acids (PUFA) were positively correlated with overweight/obesity. In contrast, monounsaturated fatty acids (MUFA) and high-quality carbohydrates were negatively correlated with overweight/obesity. In short, fat, non-high-quality protein, saturated fatty acids (SFA), and PUFA were positively correlated with the risk of obesity, whereas higher carbohydrate, MUFA, and high-quality carbohydrate intake were associated with a lower risk of obesity. Obesity can be effectively prevented by appropriately adjusting the proportion of intake from the three major macronutrients.

## 1. Introduction

Obesity is a complex chronic metabolic disease caused by the interaction of multiple factors including genetic and environmental factors, which is caused by the excessive accumulation and/or abnormal distribution of fat in the body due to excessive energy and nutrients. In the past four decades, overweight/obesity rates in China have been increasing and have reached epidemic proportions [1,2]. During this period, the number of obese adults has increased by more than four times, and the number of overweight adults has also increased by more than two times. China National Nutrition and Health Surveillance (2015–2017) shows that the obesity rate of adult residents in China increased from 7.1% in 2002 to 14.1% in 2015, and the overweight rate increased from 22.8% in 2002 to 33.3% in 2015 [3]. It is estimated that the overweight/obesity rate of adult residents in China will reach 63.5% in 2030 [4]. Obesity is the basis for diseases such as hypertension [5], diabetes [6], and stroke [7], which impose a heavy burden on the socioeconomic and healthcare systems. Therefore, preventing obesity in the population is significant for controlling the occurrence of various chronic diseases.

A rational diet is one of the key elements for the prevention of overweight/obesity. Protein, fat, and carbohydrates are the main energy nutrients for the human body and are called macronutrients. They release energy through oxidation in the body and provide energy for human life activities, also known as energy-producing nutrients. After protein is degraded into amino acids in the body, α-keto acids generated via deamination can be oxidized and decomposed directly or indirectly through the tricarboxylic acid cycle and release energy at the same time, which is one of the energy sources of the human body. Fat is an important source of energy for the human body and is the nutrient with the highest energy density in food. It can be provided by fat oxidation when the body needs energy. Dietary carbohydrates are the most economical and predominant source of energy for humans, the primary source of energy for the nervous system and myocardium, and the primary fuel for muscle activity. The oxidation of glucose in vivo can produce 16.7 kJ of energy per gram. Glycogen is the storage form of carbohydrates in muscle and the liver. The liver stores about one-third of the glycogen in the body. Glycogen in the liver can be broken down into glucose to provide energy for the body [8]. Currently, some studies have confirmed the relationship between macronutrient intake and overweight/obesity [9,10]. However, most of the studies focused on intervention experiments that were conducted to explore the weight loss effect of the energy intake from macronutrients on obese populations. Moreover, studies have seldom analyzed the relationship between energy intake from macronutrients and overweight/obesity based on the quality of macronutrients with follow-up data in large populations. Therefore, this study aimed to investigate the longitudinal relationship between the energy intake of macronutrients and overweight/obesity based on food sources.

## 2. Materials and Methods

### 2.1. Study Population

The China Health and Nutrition Survey (CHNS) is an ongoing longitudinal survey initiated in 1989, with follow-up surveys conducted in 1991, 1993, 1997, 2000, 2004, 2006, 2009, 2011, 2015, and 2018. The study design and procedures have been presented elsewhere [11]. We conducted the prospective cohort study using 10 waves of the CHNS data. We excluded participants with missing BMIs. We further excluded participants who were pregnant or lactating and who were <18 years old, and those who reported implausible energy intakes or those who were overweight/obese at baseline. The remaining participants who took part in the survey in at least 2 waves were included. Finally, a total of 14,531 participants were included in the final analysis. 

The study was conducted in accordance with the Declaration of Helsinki and approved by the Institutional Review Board of the University of North Carolina at Chapel Hill (No. 07-1963) and the Institutional Review Committee of the National Institute for Nutrition and Health, and the Chinese Center for Disease Control and Prevention approved the survey protocols, instruments, and procedures for obtaining informed consent (No. 2018-004). The approval date was 14 March 2018. Informed consent forms were signed by all respondents and their guardians prior to participation in the survey.

### 2.2. Dietary Measurements

Dietary intake was assessed by using continuous 3-day 24 h recall (2 workdays and 1 weekend day) at the individual level. The participants were asked to report the types and quantities of food and beverages they had consumed in the previous 24 h [12]. The energy and macronutrients in foods were calculated using the China food composition table. According to the Chinese Encyclopedia of Nutrition Science, high-quality protein is also called complete protein, which is defined as the complete variety, sufficient quantity, and appropriate proportion of essential amino acids. High-quality protein can not only maintain the health of adults but also promote the growth and development of children, such as milk protein, bean protein, and so on. Therefore, in this study, animal protein and soy protein were classified as high-quality proteins, and the remaining proteins were classified as non-high-quality proteins [8]. Saturated fatty acids (SFA), monounsaturated fatty acids (MUFA), and polyunsaturated fatty acids (PUFA) were the categories of fat. Based on the glycemic index and dietary fiber, we classified carbohydrates into high-quality carbohydrates and low-quality carbohydrates [13,14] (see Appendix A). Food sources constituting these categories are shown in Appendix A.

### 2.3. Other Relevant Variates

In our study, we included several confounders associated with diet and overweight/obesity via questionnaires, including age, gender (man and woman), living area (urban or rural), education level (primary and below, junior high, or senior high and above), annual household income per family member, alcohol consumption (non-current alcohol drinker or current alcohol drinker), smoking status (non-current smoker or current smoker), physical activity (PA), sedentary time (ST), and total energy intake. We included four ST domains—leisure and television time, computer time, reading time, and game time. The level of physical activity was defined as the self-reported time spent in each activity multiplied by specific metabolic equivalent values.

### 2.4. Definition of Overweight/Obesity

Trained health workers measured body weight and height based on the World Health Organization standard protocol [15,16]. In each survey, trained physicians and nurses measured height without shoes to the nearest 0.1 cm, and they measured body weight without shoes and with light clothing to the nearest 0.1 kg. We calculated BMI as weight in kg divided by height in meters (m) squared (kg/m^2^). We defined overweight as 24 kg/m^2^ ≤ BMI < 28 kg/m^2^ and obesity as a BMI ≥ 28 kg/m^2^, based on the Chinese Criteria of weight for adults (WS/T 428-2013) [17]. In this study, we defined overweight/obesity as a BMI ≥ 24 kg/m^2^.

### 2.5. Statistical Analysis

Categorical variables were expressed as percentages, while continuous variables are described as the mean (standard deviation) or median (interquartile spacing). Linear regression analysis was used to analyze the trend test for continuous variables, and the Cochran–Armitage test and Mantel–Haenszel test for trend were used for categorical variables. According to the energy intake from protein, we classified the participants into four groups: G1 (<10%), G2 (10%–<15%), G3 (15%–<20%), and G4 (≥20%). According to the energy intake from fat, we classified the participants into four groups: G1 (<20%), G2 (20%–<25%), G3 (25%–<30%), and G4 (≥30%). According to the energy intake from carbohydrates, we classified the participants into four groups: G1 (<50%), G2 (50%–<60%), G3 (60%–<65%), and G4 (≥65%). We categorized the participants into quartiles across the energy intake from subtypes of macronutrients in each survey (see Appendix B). The generalized estimating equation (GEE) model was used to analyze the association between the percentage of energy intake from macronutrients and BMI and overweight/obesity. Model 1 was a crude model. Model 2 adjusted for age, gender, living area, education level, individual income, alcohol consumption, and smoking status. Model 3 additionally adjusted for physical activity, sedentary time, total energy intake, baseline BMI, and mutual adjustments for percentages of energy intake from other specific dietary macronutrient sources. 

All analyses were carried out with SAS version 9.4 and Stata 17.0. The 2-sided *p* < 0.05 was deemed as statistically significant in all analyses.

## 3. Results

### 3.1. Descriptive Characteristics

The basic characteristics of the study population are presented in Table 1. Over the years 1991–2018, the percentage of urban participants, individual income, BMI, rate of overweight/obesity, medium to high education level, and protein (%E), fat (%E), high-quality protein (%E), SFA (%E), PUFA (%E), MUFA (%E), and high-quality carbohydrates (%E) increased over time. The percentage of men, current smokers, PA, energy intake, current alcohol drinkers, carbohydrates (%E), non-high-quality protein (%E), and low-quality carbohydrates (%E) declined over time.

### 3.2. Association between Dietary Macronutrient Intake (% of Energy) and BMI

Table 2 shows the longitudinal association between macronutrient intake (%E) and BMI. After adjusting for all potential confounders, the participants in the top group of the energy intake from fat had an increase in BMI (β = 0.21, 95% CI 0.17–0.25). The percentage of energy intake from carbohydrates was associated with a lower BMI (β = −0.22, 95% CI −0.26 to −0.18). Participants in the highest quartile of energy intake from non-high-quality protein (β = 0.06, 95% CI 0.01–0.11), SFA (β = 0.10, 95% CI 0.04–0.17), and PUFA (β = 0.12, 95% CI 0.07–0.17) were positively associated with BMI. Participants in the highest quartile of energy intake from MUFA (β = −0.13, 95% CI −0.20 to −0.06) and energy intake from high-quality carbohydrates (β = −0.11, 95% CI −0.16 to −0.07) were negatively associated with BMI.

### 3.3. Association between Dietary Macronutrient Intake (% of Energy) and Overweight/Obesity

Table 3 shows the association between energy intake from macronutrients and overweight/obesity. After adjusting for all confounders, participants in the highest group of energy intake from fat were more likely to have obesity (OR = 1.21, 95% CI 1.13–1.30). Energy intake from carbohydrates was more likely to reduce the risk of obesity (OR = 0.84 95% CI 0.78–0.90). Participants in the highest quartile of energy intake from non-high-quality protein and energy intake from PUFA were more likely to have obesity, with ORs of 1.31 (95% CI 1.20–1.43) and 1.18 (95% CI 1.09–1.28), respectively. Participants in the highest quartile of energy intake from MUFA were more likely to reduce their risk of obesity (OR = 0.83 95% CI 0.75–0.93).

## 4. Discussion

In this large-scale, 28-year follow-up study, we found that energy intake from fat was positively correlated with BMI and overweight/obesity, while energy intake from carbohydrates was negatively correlated with BMI and overweight/obesity among Chinese adults. In addition, we also found that those with a higher intake of non-high-quality protein, SFA, and PUFA had a higher BMI, and those with a higher intake of MUFA and high-quality carbohydrates had a lower BMI.

Studies on the relationship between protein intake and obesity are still contradictory. Some studies have shown that high-protein diets are beneficial to weight loss [18,19,20,21]. However, some studies have found that energy intake from protein is positively correlated with overweight/obesity. A longitudinal study in the United States found that protein intake could significantly increase the risk of overweight/obesity in men [22]. A cross-sectional study of dietary surveys of 1135 adults that examined alcohol and macronutrient intake patterns in relation to obesity and central obesity showed that protein intake was positively associated with BMI, body fat percentage, sagittal abdominal diameter, and waist circumference in men [23]. These different findings may be due to the protein source. Proteins are usually divided into animal proteins and plant proteins, which have different effects on obesity [24,25,26]. Interestingly, we found that non-high-quality protein was positively associated with obesity, which is inconsistent with previous studies that have found beneficial effects of plant protein on BMI and obesity [27,28]. The possible explanation was the different subtypes of protein. Plant proteins in previous studies included legume proteins, and we all know the beneficial effects of legumes on health [29]. In our study, the animal foods and legumes were divided into high-quality proteins based on the recommendations of dietary guidelines for Chinese residents. Most of the non-high-quality protein intake in our study was from cereal (such as wheat and rice) which was the main source of protein for Chinese adults [3]. A randomized controlled trial found that higher intake of cereal plant protein at the cost of non-cereal plant protein was associated with a larger increase in body weight [20]. It is unclear whether the effects are related to amino acid composition or other aspects of these foods. These mechanisms need to be further explored. 

In this study, we found that the energy intake from fat and PUFA was positively correlated with overweight/obesity. This result is related to the risk of obesity, with similar conclusions being reached in other studies [11,12,30,31]. Shai et al. [32] analyzed the follow-up of 121,700 adult women aged 30–55 and found that dietary fat was positively correlated with BMI. The follow-up cohort study of the China Health and Nutrition Examination Survey showed that fat intake, the percentage of energy intake from fat, and a high-fat diet were positively correlated with body weight, BMI, overweight, and obesity. The risk of fat-promoting overweight/obesity may be related to the type of fatty acid. We found that SFA was positively associated with BMI. In a cross-sectional study from seven European countries to examine cross-sectional associations with BMI and waist circumference (WC), and interaction effects of fat mass and obesity-associated gene (FTO) genotype, they found that dietary patterns with high SFA and low dietary fiber were associated with higher BMI and WC, while higher dietary fiber was inversely associated with WC among adults [33]. Diets high in SFA reduce total fat oxidation and energy expenditure and reduce diet-induced thermogenesis, which leads to fat accumulation in the body [34,35,36]. Linoleic acid (LA) and α-linolenic acid (ALA) are precursors of the *n*-6 and *n*-3 series of PUFA and have attracted much attention in recent years. Previous epidemiological studies have confirmed a positive correlation between LA/ALA intake and BMI and overweight/obesity [37,38,39]. A prospective study conducted in Germany found that the baseline levels of erythrocyte LA levels in middle-aged and older women were associated with a higher risk of overweight/obesity during a mean follow-up of 10.4 years [38]. A cross-sectional analysis, utilizing data from the National Health and Nutrition Examination Survey and the What We Eat in America study, revealed a stratified relationship between ALA intake and various sociodemographic groups. Notably, a positive correlation between ALA consumption and BMI was observed specifically within non-Hispanic black individuals [40]. Metabolites of dietary LA, such as arachidonoylethanolamide and 2-arachidonoylglycerol, reduce glucose uptake by skeletal muscle and reduce satiety signals from the hypothalamus, thereby increasing fat accumulation and promoting energy intake and weight gain. In addition, prostacyclin, which is converted from dietary LA, can promote obesity by stimulating adipocyte differentiation through a variety of pathways [35]. The obesogenic mechanism of ALA may be related to its competition with LA for the same enzymes (β-6 desaturase) [41]. In addition, frying is a common cooking method in China. Methods such as stir-frying may produce more trans-FA (TFA), thereby promoting obesity. We also found that the energy intake from MUFA was negatively correlated with overweight/obesity. Oleic acid (OA) in MUFA is a key factor in reducing the risk of overweight/obesity [39]. Diets rich in OA can increase the rate of fat oxidation compared to a high SFA diet [42]. In addition, oleoyl ethanolamide (OEA), a derivative of OA, can regulate appetite and reduce energy intake [43].

This study found that the percentage of energy intake of carbohydrates was negatively correlated with BMI, which is consistent with other studies [44,45]. A Chinese study found that higher carbohydrate intake could reduce the risk of overweight/obesity in women [46]. Conversely, other studies found a positive correlation between carbohydrate intake and BMI. The results of a randomized controlled trial in Guangdong province in China showed that a low-carbohydrate diet could reduce BMI, weight, waist circumference, waist-to-hip ratio (WHR), and body fat rate [47]. A meta-analysis also found that a low-carbohydrate diet was beneficial for weight loss in the short term [48]. It is recognized that we should emphasize the quality of carbohydrates, not just the quantity of carbohydrates. In a study in the United States, increased daily consumption of refined grains and starchy vegetables was found to be associated with long-term weight gain, whereas increased intake of whole grains, fruits, and non-starchy vegetables was associated with less weight gain [14]. In the United States Nurses’ Health Study and Health Professionals Follow-up Cohort Study, both plant-based low-carbohydrate diets and healthy low-carbohydrate diets were found to reduce body weight, and the association was stronger in young, obese, and less active populations [49]. Several mechanisms support the reduction in the risk of obesity through high-quality carbohydrates: low GI and high dietary fiber promoting satiety, reducing appetite and fat storage, increasing fat oxidation, and changing the microbiome to reduce food intake [50,51,52]. In addition, high-quality carbohydrate intake is associated with healthy lifestyles or habits, such as healthy eating patterns, higher levels of physical activity, or longer sleep durations [53,54]. 

In this study, we examined the longitudinal changes in the energy intake from macronutrients in Chinese adults over 27 consecutive years and examined the association between the energy intake from macronutrients and overweight/obesity; a large population and long-term follow-up could reduce the probability of reverse causation. In addition, we further emphasized the impact of macronutrient categories on overweight/obesity, this finding has important implications for the development of targeted nutritional intervention strategies to prevent and control obesity. Moreover, our study may provide dietary recommendations for Chinese people to prevent obesity based on their dietary patterns. The limitations of our study also need consideration. First, the 24 h dietary recall method cannot reliably reflect usual dietary intake and may generate recall bias. Second, it must be noted that the generalizability of our findings to other populations is limited. Dietary habits and cooking methods may vary considerably across cultures and regions, and these differences must be taken into account when applying our findings to other populations. In the future, we need more studies from different countries and populations to verify our results. Finally, despite our best efforts to control for known confounders, the possibility of residual confounding factors remains. This is a common limitation of observational studies and suggests that our findings should be interpreted with caution.

## 5. Conclusions

The present study findings indicated that the percentage of energy intake from subtypes of macronutrients has different effects on overweight/obesity. The “Healthy China Action (2019−2030)” and the “National Nutrition Plan (2017−2030)” both propose to slow down the rate of obesity in adults. Therefore, accurate nutrition intervention strategies about the intake of macronutrients are needed to prevent obesity among Chinese adults.

## Figures and Tables

**Table 1:** Description of the characteristics of the study population (CHNS1991-2018).

Variables	1991	1993	1997	2000	2004	2006	2009	2011	2015	2018	*p*-Trend
	(N = 5396)	(N = 5675)	(N = 5525)	(N = 6286)	(N = 6155)	(N = 6083)	(N = 6152)	(N = 6755)	(N = 7371)	(N = 6360)	
Age ^1^	41.02 ± 15.12	42.59 ± 15.43	43.82 ± 15.26	45.91 ± 14.98	48.42 ± 14.86	49.77 ± 14.67	51.24 ± 14.76	52.38 ± 14.73	53.74 ± 14.16	56.44 ± 13.91	<0.0001
Man	2758 (51.11)	2865 (50.48)	2875 (52.04)	3218 (51.19)	3055 (49.63)	2963 (48.71)	3036 (49.35)	3224 (47.73)	3372 (45.75)	2863 (45.02)	<0.0001
Urban ^2^	1655 (30.67)	1750 (30.84)	1737 (31.44)	1998 (31.78)	1926 (31.29)	1887 (31.02)	1828 (29.71)	2267 (33.56)	2561 (34.74)	2148 (33.77)	<0.0001
Education ^3^											<0.0001
Primary and below	3085 (57.24)	3088 (54.51)	2984 (54.09)	3053 (48.61)	2812 (45.71)	2697 (44.34)	2757 (44.83)	2845 (42.12)	2746 (37.25)	2221 (34.92)	
Junior high	1455 (26.99)	1602 (28.28)	1536 (27.84)	1889 (30.08)	1938 (31.50)	1880 (30.91)	2057 (33.45)	2105 (31.16)	2321 (31.49)	2055 (32.31)	
Senior high and above	850 (15.77)	975 (17.21)	997 (18.07)	1338 (21.31)	1402 (22.79)	1505 (24.75)	1336 (21.72)	1805 (26.72)	2304 (31.26)	2084 (32.77)	
Current smoker ^2^	1916 (35.51)	1902 (33.52)	1820 (32.94)	2010 (31.98)	1902 (30.90)	1745 (28.69)	1871 (30.41)	1906 (28.22)	1797 (24.38)	1349 (21.21)	<0.0001
Current alcohol drinker ^2^	2064 (38.25)	2037 (35.89)	2037 (36.87)	2225 (35.40)	2047 (33.26)	1961 (32.24)	2075 (33.73)	2216 (32.81)	2018 (27.38)	1552 (24.40)	<0.0001
Individual income	975.83 (449.55, 1305.36)	1393.89 (576.09, 1840.38)	3067.3 (1189.92, 3607.50)	3481.87 (1297.59, 4441.11)	4753.84 (1630.59, 6027.30)	5877.62 (1778.22, 7135.08)	9362.59 (3288.93, 11,555.10)	13,043.73 (4663.11, 17,032.95)	20,650.98 (5561.10, 266,31.12)	31,168.46 (6795.42, 33,921.60)	<0.0001
(RMB/year) ^1^											
PA ^1^	427.51 (199.80, 623.82)	362.42 (192.03, 512.82)	355.34 (150.96, 516.15)	291.94 (116.55, 416.25)	225.84 (61.05, 337.44)	222.35 (56.61, 326.34)	216.81 (61.61, 308.03)	208.99 (66.60, 289.71)	219.94 (59.94, 255.30)	167.91 (48.84, 220.89)	<0.0001
(METs/week)											
Energy intake	2421.35 ± 714.84	2364.14 ± 689.91	2475.5 ± 728.13	2368.49 ± 699.59	2308.21 ± 730.24	2329.47 ± 752.33	2208.1 ± 689.34	2044.51 ± 710.91	1992.91 ± 714.93	1993.1 ± 696.53	<0.0001
(kcal/day) ^1^											
BMI ^1^	20.63 ± 1.80	20.95 ± 2.02	21.26 ± 2.21	21.75 ± 2.43	21.90 ± 2.57	22.06 ± 2.61	22.14 ± 2.73	22.29 ± 2.73	22.55 ± 2.80	22.9 ± 2.92	<0.0001
Overweight (%) ^2^	-	386 (6.80)	539 (9.76)	1024 (16.29)	1208 (19.63)	1301 (21.39)	1423 (23.13)	1551 (22.96)	1852 (25.13)	2087 (32.81)	<0.0001
Protein (%E) ^4^	12.31 ± 2.47	12.56 ± 2.78	11.75 ± 2.41	11.76 ± 2.54	12.17 ± 2.77	11.88 ± 2.68	12.25 ± 2.79	12.33 ± 3.05	13.13 ± 3.44	13.11 ± 3.42	<0.0001
Fat (%E) ^4^	24.47 ± 11.55	24.51 ± 12.03	25.52 ± 12.02	28.58 ± 11.22	26.82 ± 12.34	30.52 ± 13.14	31.42 ± 11.84	34.59 ± 11.98	35.66 ± 12.32	34.9 ± 12.24	<0.0001
Carbohydrate (%E) ^4^	62.33 ± 12.44	62.05 ± 12.91	61.98 ± 12.42	58.9 ± 11.83	60.09 ± 12.58	55.22 ± 13.19	53.80 ± 11.92	52.24 ± 12.12	50.63 ± 12.61	51.41 ± 12.46	<0.0001
High-quality protein (%E) ^4^	3.80 ± 3.43	4.18 ± 3.79	4.19 ± 3.46	4.58 ± 3.48	4.89 ± 3.78	5.11 ± 3.59	5.66 ± 3.74	5.97 ± 3.94	5.92 ± 3.76	5.87 ± 3.75	<0.0001
Non-high-quality protein (%E) ^4^	8.52 ± 2.3	8.38 ± 2.55	7.56 ± 2.48	7.18 ± 2.34	7.28 ± 2.54	6.77 ± 2.39	6.59 ± 2.36	6.36 ± 2.42	7.20 ± 2.07	7.24 ± 2.14	<0.0001
SFA (%E) ^4^	5.80 ± 3.59	5.72 ± 3.66	5.51 ± 3.34	6.25 ± 3.28	5.85 ± 3.29	6.62 ± 3.37	6.54 ± 3.06	7.10 ± 3.32	6.95 ± 3.34	6.89 ± 3.39	<0.0001
MUFA (%E) ^4^	8.55 ± 4.77	8.52 ± 4.88	9.47 ± 5.58	10.01 ± 5.13	9.00 ± 5.4	10.25 ± 5.33	10.13 ± 5.06	11.61 ± 6.19	11.83 ± 6.33	11.76 ± 6.3	<0.0001
PUFA (%E) ^4^	4.15 ± 2.87	4.13 ± 2.82	5.55 ± 4.29	6.62 ± 4.46	6.44 ± 5.06	7.12 ± 4.68	7.01 ± 4.5	8.07 ± 5.06	7.70 ± 5.1	7.28 ± 4.73	<0.0001
High-quality carbohydrates (%E) ^4^	7.78 ± 11.11	8.03 ± 11	6.9 ± 8.69	6.61 ± 7.23	6.74 ± 7.29	6.07 ± 5.87	6.97 ± 6.33	8.13 ± 6.61	7.97 ± 6.86	8.26 ± 7.47	<0.0001
Low-quality carbohydrates (%E) ^4^	54.55 ± 14.61	54.02 ± 14.95	55.08 ± 13.62	52.29 ± 12.34	53.34 ± 13.64	49.15 ± 14.06	46.83 ± 12.98	44.11 ± 13.05	42.66 ± 13.36	43.15 ± 13.22	<0.0001

^1^ Linear regression analysis was applied; ^2^ the Cochran–Armitage test was applied; ^3^ the Mantel–Haenszel test was applied; ^4^ the linear trend test was applied. The models were adjusted for age, gender, urban and rural areas, education level, smoking status, alcohol consumption, individual income, ST, PA, total energy, and BMI.

**Table 2:** Regression coefficients (95% CI) of BMI according to energy intake from macronutrients ^†^.

	G1/Q1	G2/Q2	G3/Q3	G4/Q4
Protein (%E)				
Model 1	0	−0.05 (−0.09, −0.01) *	0.19 (0.13, 0.25) ***	0.25 (0.13, 0.38) ***
Model 2	0	−0.01 (−0.05, 0.03)	0.05 (−0.01, 0.11)	0.06 (−0.06, 0.18)
Model 3	0	0.00 (−0.04, 0.04)	0.06 (0.01, 0.11) *	0.05 (−0.06, 0.17)
Fat (%E)				
Model 1	0	0.22 (0.17, 0.28) ***	0.40 (0.35, 0.45) ***	0.65 (0.60, 0.70) ***
Model 2	0	0.04 (−0.01, 0.09)	0.12 (0.07, 0.16) ***	0.20 (0.16, 0.24) ***
Model 3	0	0.05 (0.01, 0.10) *	0.13 (0.08, 0.17) ***	0.21 (0.17, 0.25) ***
Carbohydrates (%E)				
Model 1	0	−0.25 (−0.29, −0.21) ***	−0.49 (−0.54, −0.44) ***	−0.74 (−0.79, −0.69) ***
Model 2	0	−0.01 (−0.02, −0.01) ***	−0.02 (−0.03, −0.02) ***	−0.03 (−0.04, −0.03) ***
Model 3	0	−0.08 (−0.11, −0.04) ***	−0.16 (−0.21, −0.12) ***	−0.22 (−0.26, −0.18) ***
High-quality protein (%E)				
Model 1	0	0.02 (−0.03, 0.07)	0.03 (−0.02, 0.08)	0.07 (0.01, 0.12) *
Model 2	0	0.02 (−0.02, 0.06)	0.04 (−0.01, 0.08)	0.06 (0.01.0.11) *
Model 3	0	0.00 (−0.04, 0.04)	0.00 (−0.05, 0.05)	0.00 (−0.05, 0.06)
Non-high-quality protein (%E)				
Model 1	0	−0.06 (−0.10, −0.02) **	−0.06 (−0.11, −0.01) *	−0.04 (−0.09, 0.01)
Model 2	0	−0.05 (−0.09, −0.01) *	−0.04 (−0.09, −0.01) *	−0.04 (−0.09, 0.01)
Model 3	0	0.00 (−0.04, 0.04)	0.03 (−0.02, 0.08)	0.06 (0.01, 0.11) *
SFA (%E)				
Model 1	0	−0.01 (−0.06, 0.03)	0.03 (−0.02, 0.08)	0.07 (0.02, 0.12) **
Model 2	0	−0.02 (−0.06, 0.02)	0.02 (−0.03, 0.06)	0.05 (0.01, 0.10) *
Model 3	0	0.00 (−0.05, 0.04)	0.05 (−0.01, 0.11)	0.10 (0.04, 0.17) ***
MUFA (%E)				
Model 1	0	−0.01 (−0.06, 0.03)	0.01 (−0.03, 0.06)	0.04 (−0.01, 0.09)
Model 2	0	−0.02 (−0.06, 0.02)	−0.02 (−0.06, 0.03)	0.01 (−0.04, 0.05)
Model 3	0	−0.07 (−0.12, −0.02) **	−0.11 (−0.17, −0.05) ***	−0.13 (−0.20, −0.06) ***
PUFA (%E)				
Model 1	0	0.02 (−0.02, 0.07)	0.08 (0.03, 0.12) **	0.13 (0.08, 0.18) ***
Model 2	0	0.02 (−0.02, 0.06)	0.06 (0.02, 0.10) **	0.10 (0.06, 0.15) ***
Model 3	0	0.04 (−0.01, 0.08)	0.09 (0.05, 0.14) ***	0.12 (0.07, 0.17) ***
High-quality carbohydrates (%E)				
Model 1	0	0.02 (−0.03, 0.06)	−0.03 (−0.07, 0.01)	−0.05 (−0.010, 0.01)
Model 2	0	0.02 (0.02, 0.06) *	−0.02 (−0.06, 0.02)	−0.03 (−0.08, 0.01)
Model 3	0	0.01 (−0.03, 0.05)	−0.04 (−0.08, −0.01) *	−0.11 (−0.16, −0.07) ***
Low-quality carbohydrates (%E)	0			
Model 1	0	−0.00 (−0.05, 0.04)	−0.02 (−0.06, 0.03)	−0.02 (−0.07, 0.03)
Model 2	0	0.00 (−0.04, 0.04)	0.00 (−0.04, 0.04)	−0.01 (−0.05, 0.04)
Model 3	0	−0.01 (−0.05, 0.03)	−0.01 (−0.05, 0.03)	0.02 (−0.03, 0.06)

* *p* < 0.05, ** *p* < 0.01, and *** *p* < 0.001. ^†^ G1–G4 are the groups of energy intake from protein/fat/carbohydrates, and Q1–Q4 are the quartiles of different categories of energy intake from macronutrients.

**Table 3:** ORs (95% CI) of overweight/obesity across energy intake from macronutrients ^†^.

	G1/Q1	G2/Q2	G3/Q3	G4/Q4
Protein (%E)				
Model 1	1	0.95 (0.90, 1.01)	1.24 (1.16, 1.33) ***	1.22 (1.04, 1.42) *
Model 2	1	1.12 (1.06, 1.19) ***	1.20 (1.11, 1.30) ***	1.08 (0.92, 1.27)
Model 3	1	1.12 (1.05, 1.19) ***	1.20 (1.10, 1.30) ***	1.04 (0.96, 1.24)
Fat (%E)				
Model 1	1	1.32 (1.22, 1.44) ***	1.57, 1.45, 1.70) ***	1.99 (1.86, 2.13) ***
Model 2	1	1.06 (0.98, 1.14)	1.16 (1.07, 1.25) ***	1.25 (1.17, 1.34) ***
Model 3	1	1.05 (0.96, 1.14)	1.13 (1.04, 1.22) **	1.21 (1.13, 1.30) ***
Carbohydrates (%E)				
Model 1	1	0.80 (0.76, 0.84) ***	0.63 (0.58, 0.67) ***	0.47 (0.44, 0.50) ***
Model 2	1	0.97 (0.92, 1.02)	0.86 (0.80, 0.93) ***	0.79 (0.74, 0.85) ***
Model 3	1	1.00 (0.95, 1.06)	0.89 (0.82, 0.96) **	0.84 (0.78, 0.90) ***
High-quality protein (%E)				
Model 1	1	1.04 (0.97, 1.10)	1.05 (0.98, 1.12)	1.04 (0.97, 1.12)
Model 2	1	1.03 (0.97, 1.10)	1.04 (0.97, 1.12)	1.02 (0.94, 1.10)
Model 3	1	1.05 (0.98, 1.13)	1.08 (0.99, 1.16)	1.09 (0.99, 1.18)
Non-high-quality protein (%E)				
Model 1	1	0.97 (0.91, 1.03)	1.00 (0.94, 1.07)	1.02 (0.95, 1.09)
Model 2	1	1.00 (0.94, 1.06)	1.04 (0.98, 1.12)	1.18 (1.09, 1.27) ***
Model 3	1	1.07 (1.01, 1.15) *	1.15 (1.07, 1.24) ***	1.31 (1.20, 1.43) ***
SFA(%E)				
Model 1	1	0.99 (0.93, 1.05)	1.03 (0.96, 1.09)	1.06 (0.99, 1.13)
Model 2	1	0.96 (0.91, 1.02)	0.98 (0.92, 1.04)	0.97 (0.91, 1.04)
Model 3	1	0.98 (0.91, 1.05)	1.04 (0.95, 1.12)	1.09 (0.98, 1.21)
MUFA (%E)				
Model 1	1	0.98 (0.92, 1.04)	0.99 (0.93, 1.06)	1.01 (0.94, 1.08)
Model 2	1	0.92 (0.87, 0.98) *	0.93 (0.87, 0.99) *	0.92 (0.86, 0.98) *
Model 3	1	0.90 (0.83, 0.97) **	0.88 (0.80, 0.96) **	0.83 (0.75, 0.93) **
PUFA (%E)				
Model 1	1	0.98 (0.92, 1.05)	1.05 (0.99, 1.12)	1.13 (1.06, 1.21) ***
Model 2	1	1.01 (0.95, 1.07)	1.09 (1.02, 1.16) **	1.23 (1.15, 1.32) ***
Model 3	1	1.04 (0.97, 1.11)	1.08 (1.04, 1.12) **	1.18 (1.09, 1.28) ***
High-quality carbohydrates (%E)				
Model 1	1	1.02 (0.96, 1.08)	0.97 (0.92, 1.03)	0.95 (0.90, 1.02)
Model 2	1	1.05 (0.99, 1.11)	1.01 (0.95, 1.07)	1.05 (0.98, 1.12)
Model 3	1	1.04 (0.97, 1.10)	0.98 (0.92, 1.04)	0.95 (0.88, 1.02)
Low-quality carbohydrates (%E)				
Model 1	1	1.04 (0.98, 1.10)	1.00 (0.94, 1.06)	0.99 (0.93, 1.06)
Model 2	1	1.08 (1.01, 1.14) *	0.99 (0.93, 1.05)	0.93 (0.87, 1.01) *
Model 3	1	1.10 (1.03, 1.18) **	1.03 (0.96, 1.10)	0.99 (0.91, 1.07)

* *p* < 0.05, ** *p* < 0.01, and *** *p* < 0.001. ^†^ G1–G4 are the groups of energy intake from protein/fat/carbohydrate, and Q1–Q4 are the quartiles of different categories of energy intake from macronutrients.

## Data Availability

The datasets generated and analyzed during the current study are available from the corresponding author (J.Z.) upon reasonable request.

## Appendix A

**Table A1:** Dietary components classified as food sources of carbohydrates and protein.

Food Groups	Subcategories	Items
High-quality carbohydrate	Whole grain	Maize, barley, millet, sorghum, buckwheat, pearl barley, and pros millet
	Fruit	Any fresh, canned, and dried fruits
	Non-starchy vegetables	Greens, crown daisy, broccoli, cucumber, peppers, onions, tomatoes, asparagus, carrots, sweet potatoes, pumpkin, and winter squash
	Legumes	Soybean, tofu or other soy protein, peas, and other legumes
	Algae	
Low-quality carbohydrate	Starchy vegetables	Potatoes, dasheen, and yam
	Refined grain	White rice, white wheat, white wheat power, etc.
	Added sugars	Sugar, candy, preserved fruit, sugar-sweetened beverages, brownies, ice cream, and cake
	Other	All other carbohydrates not included in the groups above
High-quality protein	Poultry	Chicken meat and duck meat
	Red meat	Pork, beef, and mutton
	Egg	Eggs, duck eggs, quail eggs, etc.
	Dairy products	Whole milk, skim milk, cheese, and yogurt
	Seafood	Fish, shrimp, and shellfish
	Legumes	As above
Non-high-quality protein	Grain	As above
	Nuts	Peanuts, walnuts, and other nuts
	Other	All other proteins not included in the specific groups above

## Appendix B

**Table A2:** Grouping of macronutrient categories by survey year.

Year	High-Quality Protein (%E)	Non-High-Quality Protein (%E)	SFA (%E)	MUFA (%E)	MUFA (%E)	High-Quality Carbohydrate (%E)	Low-Quality Carbohydrate (%E)
1991							
Q1	0.00 (0.00, 1.04)	5.86 (0.14, 6.89)	2.06 (0.00, 3.13)	3.20 (0.00, 4.99)	1.22 (0.00, 1.95)	1.52 (0.00, 2.19)	39.57 (0.03, 46.47)
Q2	2.02 (1.05, 3.07)	7.81 (6.89, 8.60)	4.21 (3.13, 5.20)	6.64 (5.00, 8.08)	2.76 (1.95, 3.63)	2.91 (2.19.3.82)	51.73 (46.47, 56.24)
Q3	4.32 (3.07, 5.72)	9.36 (8.60, 10.12)	6.35 (8.60, 10.12)	9.56 (8.08, 11.50)	4.55 (3.63, 5.68)	5.14 (3.82, 7.59)	60.29 (56.24, 64.65)
Q4	7.91 (5.72, 25.23)	11.12 (10.13, 18.44)	9.86 (7.69, 28.39)	13.99 (11.50, 36.85)	7.51 (5.68, 23.29)	15.56 (7.59, 77.02)	69.88 (64.65, 88.01)
1993							
Q1	0.00 (0.00, 1.22)	5.63 (0.20, 6.73)	1.91 (0.00, 3.08)	2.95 (0.50, 0.03)	1.13 (0.00, 1.91)	1.62 (0.20, 0.35)	37.96 (0.11, 46.24)
Q2	2.36 (1.22, 3.44)	7.54 (6.73, 8.26)	4.15 (3.08, 5.16)	6.72 (5.03, 8.08)	2.83 (1.91, 3.74)	3.16 (2.35.4.13)	51.49 (46.25, 55.93)
Q3	4.67 (3.44, 6.22)	9.05 (8.26, 9.90)	6.20 (8.60, 10.13)	9.55 (8.08, 11.41)	4.74 (3.74, 5.81)	5.57 (4.13, 8.52)	59.92 (55.94, 64.07)
Q4	8.49 (6.22, 35.48)	11.22 (9.90, 28.07)	9.81 (7.57, 30.84)	13.96 (11.41, 36.75)	7.24 (5.81, 22.61)	15.55 (8.52, 90.15)	69.72 (64.09, 87.78)
1997							
Q1	0.26 (0.00, 1.39)	4.76 (0.04, 5.82)	2.10 (0.00, 3.08)	3.40 (0.00, 5.41)	1.54 (0.00, 2.58)	1.48 (0.00, 2.2)	39.67 (0.17, 46.75)
Q2	2.53 (1.39, 3.66)	6.66 (5.82, 7.43)	4.13 (3.08, 5.07)	7.19 (5.41, 8.84)	3.60 (2.58, 4.67)	2.86 (2.20, 3.75)	52.08 (46.77, 56.2)
Q3	4.82 (3.66, 6.20)	8.27 (7.44, 9.20)	6.03 (8.60, 10.14)	10.53 (8.84, 12.63)	5.79 (4.67, 7.13)	5.12 (3.75, 8.11)	60.13 (56.20, 64.32)
Q4	8.2 (6.21, 23.06)	10.58 (9.2, 16.62)	9.12 (7.16, 34.14)	15.71 (12.63, 46.28)	9.88 (7.14, 39.58)	14.15 (8.11, 80.17)	70.26 (64.33, 86.51)
2000							
Q1	0.61 (0.00, 1.88)	4.64 (0.06, 5.61)	2.71 (0.00, 3.87)	4.38 (0.00, 6.28)	2.25 (0.30, 0.45)	1.70 (0.00, 2.37)	38.01 (0.33, 44.35)
Q2	3.01 (1.88, 4.08)	6.31 (5.62, 7.02)	4.86 (3.87, 5.87)	7.96 (6.29, 9.55)	4.63 (3.45, 5.67)	3.09 (2.00, 37.40)	48.94 (44.36, 52.83)
Q3	5.25 (4.08, 6.64)	7.79 (7.02, 8.69)	6.82 (8.60, 10.15)	11.2 (9.56, 12.88)	6.83 (5.67, 8.60)	5.45 (4.00, 7.94)	56.69 (52.83, 60.84)
Q4	8.74 (6.64, 20.22)	9.94 (8.69, 26.12)	10.04 (8.11, 27.44)	15.5 (12.88, 48.64)	11.67 (8.6, 39.09)	13.19 (7.95, 75.77)	66.55 (60.84, 85.76)
2004							
Q1	0.74 (0.00, 2.02)	4.49 (0.11, 5.60)	2.08 (0.00, 3.38)	3.08 (0.00, 5.18)	1.61 (0.00, 2.81)	1.79 (0.00, 2.52)	37.80 (1.60, 44.64)
Q2	3.11 (2.02, 4.26)	6.40 (5.60, 7.13)	4.52 (3.39, 5.56)	6.90 (5.18, 8.50)	3.98 (2.82, 5.32)	3.32 (2.52.4.32)	49.69 (44.6754.16)
Q3	5.51 (4.26, 7.04)	7.89 (7.14, 8.82)	6.60 (8.60, 10.16)	10.03 (8.50, 11.97)	6.70 (5.33, 8.65)	5.80 (4.32, 8.13)	58.35 (54.16, 63.02)
Q4	9.31 (7.04, 29.67)	10.19 (8.82, 20.09)	9.68 (7.83, 24.19)	14.92 (11.97, 39.17)	12.19 (8.65, 39.23)	13.04 (8.13, 78.03)	68.71 (63.03, 85.57)
2006							
Q1	1.25 (0.00, 2.44)	4.14 (0.050.5.16)	2.93 (0.00, 4.17)	4.30 (0.00, 6.32)	2.43 (0.00, 3.75)	1.73 (0.00, 2.4)	32.69 (0.68, 39.82)
Q2	3.47 (2.44, 4.57)	5.93 (5.16, 6.64)	5.22 (4.17, 6.22)	8.12 (6.32, 9.76)	5.00 (3.76, 6.23)	3.13 (2.40, 4.04)	44.87 (39.83, 49.56)
Q3	5.78 (4.58, 7.09)	7.38 (6.64, 8.28)	7.27 (8.60, 10.17)	11.49 (9.76, 13.38)	7.59 (6.23, 9.27)	5.50 (4.04, 7.87)	54.13 (49.57, 59.14)
Q4	9.23 (7.09, 24.41)	9.38 (8.28, 21.58)	10.32 (8.55, 30.7)	16.17 (13.38, 41.64)	12.18 (9.28, 34.91)	11.74 (7.87, 77.69)	66.03 (59.14, 87.4)
2009							
Q1	1.61 (0.00, 2.87)	3.95 (0.06, 5.01)	3.24 (0.00, 4.44)	4.64 (0.00, 6.51)	2.18 (0.00, 3.68)	2.02 (0.00, 2.86)	31.54 (3.29, 38.1)
Q2	4.05 (2.88, 5.11)	5.78 (5.01, 6.45)	5.33 (4.44, 6.27)	8.04 (6.51, 9.48)	5.02 (3.68, 6.27)	3.79 (2.86.4.93)	42.93 (38.10, 47.26)
Q3	6.32 (5.11, 7.81)	7.20 (6.45, 8.07)	7.17 (8.60, 10.18)	11.09 (9.49, 3.05)	7.63 (6.27, 9.40)	6.60 (4.93, 8.74)	51.52 (47.26, 55.77)
Q4	10.02 (7.81, 33.66)	9.38 (8.07, 20.63)	9.89 (8.21, 27.55)	16.16 (13.06, 41.64)	12.34 (9.4, 32.88)	12.95 (8.75, 63.84)	61.81 (55.80, 83.88)
2011							
Q1	1.69 (0.30, 0.09)	3.65 (0.00, 4.75)	3.60 (0.00, 4.80)	5.28 (0.00, 7.24)	3.13 (0.00, 4.73)	2.32 (0.00, 3.47)	28.90 (2.21, 34.72)
Q2	4.23 (3.09, 5.39)	5.61 (4.75, 6.30)	5.81 (4.80, 6.74)	9.03 (7.24, 10.68)	5.83 (4.74, 6.99)	4.73 (3.47.6.24)	39.56 (34.73, 43.85)
Q3	6.67 (5.39, 8.18)	7.02 (6.30, 7.86)	7.71 (8.60, 10.19)	12.54 (10.68, 14.93)	8.40 (6.99, 10.45)	8.02 (6.24, 10.77)	48.13 (43.86, 53.29)
Q4	10.69 (8.18, 29.14)	8.95 (7.86, 35.41)	10.76 (8.98, 27.39)	18.16 (14.93, 52.36)	13.82 (10.46, 53.14)	15.49 (10.77, 57.72)	59.84 (53.3, 87.39)
2015							
Q1	1.66 (0.00, 3.13)	4.94 (1.23, 5.79)	3.34 (0.00, 4.68)	5.22 (0.00, 7.44)	2.80 (0.00, 4.20)	2.07 (0.00, 3.23)	26.99 (1.43, 33.49)
Q2	4.34 (3.13, 5.48)	6.46 (5.79, 7.03)	5.70 (4.68, 6.69)	9.21 (7.45, 10.82)	5.43 (4.20, 6.56)	4.47 (3.0024.60)	38.35 (33.49, 42.47)
Q3	6.75 (5.48, 8.19)	7.71 (7.03, 8.46)	7.62 (8.60, 10.20)	12.69 (10.83, 15.12)	8.07 (6.56, 10.06)	7.85 (6.00, 10.42)	46.59 (42.48, 51.55)
Q4	10.36 (8.19, 29.11)	9.59 (8.46, 20.10)	10.49 (8.84, 30.50)	19.13 (15.12, 47.15)	13.08 (10.06, 44.13)	15.14 (10.42, 65.98)	58.27 (51.56, 86.29)
2018							
Q1	1.71 (0.00, 3.12)	4.87 (0.97, 5.75)	3.29 (0.04, 4.53)	5.11 (0.04, 7.11)	2.62 (0.04, 4.01)	1.82 (0.00, 3.07)	28.17 (1.42, 34.06)
Q2	4.22 (3.13, 5.38)	6.46 (5.79, 7.03)	5.56 (4.53, 6.48)	8.99 (7.11, 10.77)	5.19 (4.02, 6.36)	4.37 (3.07.6.02)	38.48 (34.06, 42.81)
Q3	6.56 (5.38, 8.02)	7.78 (7.10, 8.60)	7.53 (8.60, 10.21)	12.87 (10.78, 15.36)	7.68 (6.37, 9.42)	8.17 (6.02, 11.10)	47.39 (42.81, 51.97)
Q4	10.21 (8.02, 28.05)	9.65 (8.60, 22.90)	10.75 (8.78, 26.57)	19.05 (15.36, 53.75)	12.31 (9.42, 47.10)	16.01 (11.11, 70.69)	58.52 (51.98, 93.72)
